# Proteoglycans—Biomarkers and Targets in Cancer Therapy

**DOI:** 10.3389/fendo.2018.00069

**Published:** 2018-03-06

**Authors:** Dragana Nikitovic, Aikaterini Berdiaki, Ioanna Spyridaki, Theodoros Krasanakis, Aristidis Tsatsakis, George N. Tzanakakis

**Affiliations:** ^1^Laboratory of Anatomy-Histology-Embryology, Medical School, University of Crete, Heraklion, Greece; ^2^Laboratory of Toxicology, Medical School, University of Crete, Heraklion, Greece

**Keywords:** proteoglycans, cancer biology, biomarkers, molecular signature, cytokine signaling

## Abstract

Proteoglycans (PGs), important constituents of the extracellular matrix, have been associated with cancer pathogenesis. Their unique structure consisting of a protein core and glycosaminoglycan chains endowed with fine modifications constitutes these molecules as capable cellular effectors important for homeostasis and contributing to disease progression. Indeed, differential expression of PGs and their interacting proteins has been characterized as specific for disease evolvement in various cancer types. Importantly, PGs to a large extent regulate the bioavailability of hormones, growth factors, and cytokines as well as the activation of their respective receptors which regulate phenotypic diversibility, gene expression and rates of recurrence in specific tumor types. Defining and targeting these effectors on an individual patient basis offers ground for the development of newer therapeutic approaches which may act as either supportive or a substitute treatment to the standard therapy protocols. This review discusses the roles of PGs in cancer progression, developing technologies utilized for the defining of the PG “signature” in disease, and how this may facilitate the generation of tailor-made cancer strategies.

## Introduction

Proteoglycans (PGs) are composite molecules in which a glycosaminoglycan (GAG) chain(s) is covalently bound into a protein core. PGs, produced by, practically all, mammalian cells can be embedded into the plasma membranes, associated to the plasma membrane (pericellular), secreted into the extracellular matrix (ECM proper), or stored in secretory granules (intracellular). The enlistment of PGs to above classes is based on the following criteria: cellular and subcellular location, overall gene/protein homology, and the use of specific protein modules within their respective protein cores. It must, however, be mentioned, that there is a great deal of overlapping among the classes (1–3). We can certainly start with the statement that PGs have an important role in the formation of the ECM super-assembly as well as in determining of its physicochemical properties (1). Additionally, it can be said that PGs are receptors of a varying sensitivity and serve as a reservoir of biologically active mediators including growth factors (4, 5). Noteworthy, a single cell type can produce many different PG types. Osteoblast-derived cells for example secrete various ECM PGs and express on their membranes a number of cell-surface PGs (2, 6). Approximately 45 PGs have been identified to date, but each of the PGs members exhibits enormous variability (3). This inherent variability is determined by protein core modifications and by the number, type, and fine structural modifications of its GAG chains as well as by the different stoichiometry of GAG chain substitution (1, 3, 7). Briefly, PGs may have a number of GAG chain attachment sites which are not utilized equally or may exist as part-time PGs being expressed in both substituted and non-substituted forms. Furthermore, the fine structural modifications of PGs are tissue type and cell type specific. Therefore, each PG, as defined by its core protein, actually stands for a varying population of molecules where each “variant” represents a discrete structural entity (1, 3).

## Main PG Types

All PGs in respect to their cell location, as mentioned above, can be classified into intracellular, pericellular, ECM proper, and intracellular PGs. The unique intracellular PG serglycin (8) which is mostly involved in inflammatory reactions presents a category on its own. Pericellular PGs, heparan sulfate (HS)-carrying perlecan and agrin as well as Collagens XVIII and XV are mostly involved in the assembly of basement membranes. The largely HS-decorated, cell-surface PGs consist of the seven transmembrane members, e.g., syndecans 1–4 and the six GPI-anchored glypicans (1, 9, 10). Syndecans have important roles in integrin, growth factor, and growth factor receptor signaling (10, 11) whereas glypicans (12) are capable of regulating key cellular signaling pathways including Hedgehog signaling (9) and Wnt signaling (13).

The ECM proper PGs consist of 25 members: the four hyaluronan-binding hyalectans, key structural components of cartilage, blood vessels, and central nervous systems; the 18 small leucine-rich proteoglycans (SLRPs) and the three calcium-binding HSPGs, testicans (1). As regarding hyalectins, early studies demonstrated that these chondroitin sulfate (CS) PGs contain lectin-like and growth factor-like sequences (14, 15), SLRPs are modulators of ligand–receptor binding (2), autophagy regulators, and damage associated molecular patterns (16).

## PGs in Cancer

Proteoglycans contribute to cancer pathogenesis. Malignant tumors have individual PG profiles, which are closely associated with their differentiation and biological behavior, mesenchymal tumors showing a different profile from that of epithelial tumors (17, 18). Indeed, it was recently discussed that the role of the HSPG, syndecan-2 in cancer pathogenesis is conditional on cancer tissue origin determining its use as a biomarker/therapeutic target feasible (18). Noteworthy, GAG/PG effects are decidedly contingent on the specific correlation among their localization, expression, and disease type/stage. The unique, as regarding its localization, intracellular PG, serglycin has first been identified in hemopoietic cells (19). A number of later studies indicated its role in the process of malignant transformation of hemopoitic cells culminating with the suggestion that the malignant transformation of lymphoid cells may be characterized by, among other, increased synthesis of serglycin (20). In continuation, higher expression of serglycin was shown in aggressive lung, colon, and breast as well as prostate cancer cell lines (21). Furthermore, serglycin expression has been correlated to highly metastatic nasopharyngeal (22) and hepato-cellular carcinoma (HCC) metastasis to bone (23). Interestingly, it was demonstrated that serglycin has an obligatory participation in multiple myeloma cell *in vivo* growth and adhesion as well as vascularization (24).

The basement membrane PG, perlecan was initially implicated in cancer pathogenesis in a liver cancer mouse model (25). Further studies, among other by the Kovalszky group, demonstrated that this PG is not expressed by liver parenchyma tissue but rather to tumor blood vessel walls (26). Likewise, early studies demonstrated an increased expression of perlecan in the pericellular matrix of metastatic melanoma tumor samples (27). In continuation it was demonstrated that suppression of perlecan downregulates invasive behavior of melanoma cells (28) and blocks tumor growth and angiogenesis *in vivo* (29).

The hyalectan versican was demonstrated to be overexpressed in a range of cancers, including prostate, malignant myeloma, breast, glioblastoma, laryngeal, ovarian, pancreatic, cervical, gastric, and testicular germ-cell cancer as reviewed by Binder et al. (30). Five isoforms of versican, with various GAG-binding abilities and signaling properties, are generated through alternative splicing (31). The role of other hyalectans in cancer is not well established, even though evidence shows some contribution. Thus, the hyalectan aggrecan is postulated as tumor suppressor as decreased aggrecan expression correlates with metastasis and poor prognosis in laryngeal cancer (32). As regarding SLRPs, there is an exquisite specificity of discrete members’ role in carcinogenesis (2). Thus, decorin is a strong tumor suppressor (33) whereas, the expression of lumican needs to be specifically correlated to the tumor type and stage during the disease progression to draw more relevant conclusions (7). On the other hand, biglycan, mostly due to its inflammation-regulatory roles has mostly a tumor-promoting function (34). Immediate correlation between cell membrane PGs and cancer pathogenesis has been established. Thus, glypican-3 (GPC3) has emerged as a candidate therapeutic target in HCC, involving cell-cycle arrest at G1 phase through Yes-associated protein signaling (35). Importantly, GPC3 is not expressed by normal hepatic tissue but is expressed during the process of malignant transformation (36). On the other hand, downregulation of GPC3 expression in a mouse model facilitated ovarian cancer cell tumorigenicity (37).

## PGs Regulate Cytokine Signaling in Cancer Tissues

Importantly, PGs can regulate the bioavailability of hormones, growth factors, and cytokines as well as the activation of their respective receptors (38), which affects phenotypic diversibility, gene expression, and rates of recurrence in specific tumor types (39, 40). To the cell membrane syndecans, was annotated the archetypal role in regulating basic fibroblast growth factor-dependent (FGF-2) signaling (41). Subsequently, the ability of syndecans to bind and to present growth factors to their respective growth factor receptors was associated with specific cell functions. Thus, it was shown that the inhibition of myogenic differentiation by FGF-1, FGF-2 as well as Kaposi’s sarcoma FGFs was directly correlated to cellular HS (42). Indeed, to this PG family was annotated the ability to regulate the signaling properties of GFs endowed with the capability to bind heparin. Indeed, to syndecan-1 was attributed the ability to act as a functional co-receptor for hepatocyte growth factor (HGF) that induces the initiation of the receptor tyrosine kinase for HGF (Met). This tyrosine kinase enhances cell viability and growth of multiple myeloma cells through phosphatidylinositol 3-kinase/protein kinase B and RAS/mitogen-activated protein kinase pathways (43). Recent studies, present the exquisite specificity of syndecans signaling in cancer (10, 18). Thus, syndecan-2 was found to regulate a key transforming growth factor beta-2/Smad2-signaling axis in fibrosarcoma adhesion (44). Furthermore, this syndecan was shown to propagate IGF-I/IGF-IR signaling through ezrin/erk downstream activation (45). Anti-GPC3 therapy was found to dowregulate insulin-like growth factor-II expression and downstream signaling involving downregulation of HSPGs, deactivation of sulfase-2 as well as a decrease of caspase-3 gene expression. This was strongly correlated to attenuation of HCC progression (46).

**Figure 1:**
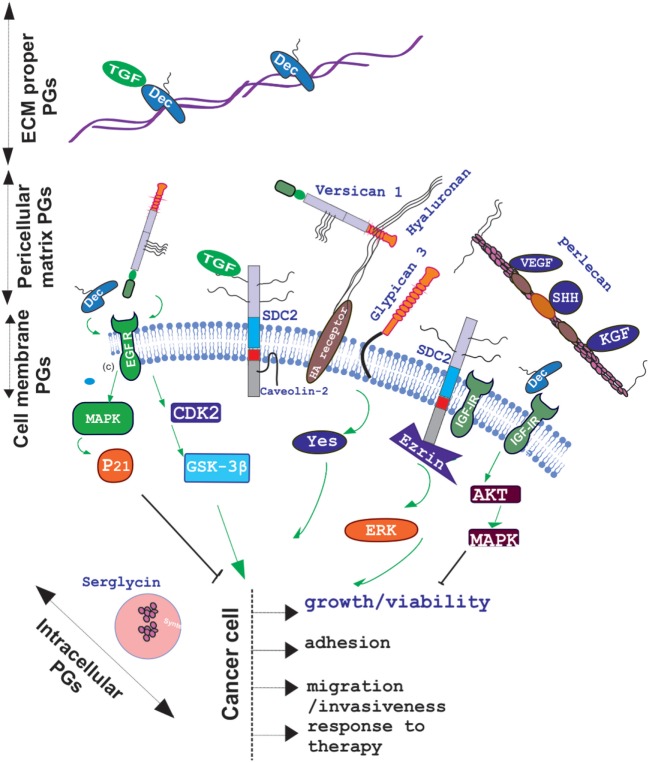
Schematic presentation of proteoglycan (PG) localization and regulation of cancer relevant cytokine signaling. PGs are located to the extracellular matrix (ECM) proper, pericellular matrix, cell membrane, or intracellular granules. Decorin (Dec), bound in to collagen fibers to the ECM specifically binds growth factors including TGF to create ECM “pools”; whereas pericellular decorin binds to EGFR and/or IGFR to attenuate their downstream signaling and induce growth arrest. Versican binds to through its EFG motif to EGFR and facilitates cancer cell growth, migration, and invasion in a CDK2/GSK-3β-dependent manner whereas through hyaluronan–hyaluronan receptor interaction it regulates cancer progression in a positive or negative manner depending on the context. Pericellular matrix, perlecan regulates VEGF, SHH, KGF, Flt-1, and Flk-1 bioavailability to affect cancer progression. Cell membrane, syndecan-2 was found to regulate, in fibrosarcoma, a transforming growth factor beta-2 (TGF-β2)/Smad2-signaling axis and to propagate IGF-I/IGF-IR signaling through ezrin/erk downstream activation.

The role of other PG classes in cytokine signaling mediation has been highlighted in numerous studies. Recently, the opposite role of decorin and versican in the regulation of the cross talk between estradiol receptor and EGFR/IGF-IR signaling pathways in estrogen-responsive breast cancers have been discussed (47). Specifically, higher decorin expression in the tumor stroma in node-negative invasive breast cancer is correlated to better prognosis (48) due to the modulation of the EGF family of tyrosine kinase receptors downstream signaling (49). This complex mechanism of decorin action starts with its binding to the EGFR which results in receptor dimerization and protracted phosphorylation of the downstream MAP kinase (50, 51). The cyclin-dependent kinase inhibitor p21WAF1/Cip1 (p21) is thereupon induced which downregulates tumor growth (52). The binding of decorin to EGFR, however, ultimately results in receptor degradation *via* sustained internalization, executed through caveolar-dependent endocytosis (50). On the other hand, Du et al., showed that the versican G3 domain facilitated breast cancer cell proliferation, mobility, and capability to metastasize to distant sites by enhancing signaling pathways dependent on EGFR activation (53). Specifically, the same authors show that the CDK2 and GSK-3β (S9P) actions dependent on the EGFR/ERK signaling decreased the apoptosis of these cells. Moreover, versican G3 expressing breast cancer cells had a poorer response to chemotherapeutic drugs treatments, including doxorubicin or epirubicin (54). The role of decorin in IGF-IR activation and endocytisis has likewise been analyzed in several cancer models (55). In addition, it was demonstrated that downregulated decorin expression facilities the process of hepatic carcinogenesis *in vivo* by permitting PDGFRα, EGFR, IGF-IR, and MSPR/RON tumorigenic signaling (56). The basal membrane perlecan and its binding ligands, including VEGF, SHH, KGF, Flt-1, and Flk-1, were found to be differentially expressed in oral epithelial dysplasia and carcinoma *in situ* indicating to the important contribution of perlecan in the regulation of these factors signaling (57). The effect of PGs on cytokine signaling in cancer and the resulting action on cancer cell function is schematically depicted in Figure 1.

## Strategies Focused on Defining PG Molecular Signature in Cancer Progression

Due to their key roles in tumorigenesis a number of methods has evolved to determine PG “molecular signature” in cancer. Thus, a combination of comparative genomic expression profiling and immunohistochemical staining of tissue microarrays from patients followed by multivariate analyses exhibited serglycin as an unfavorable independent indicator of distant metastasis-free and disease-free survival (22). This allowed the characterization of serglycin as an independent prognostic indicator of metastasis-free survival and disease-free survival in patients. An innovative approach was the utilization of Matrix-assisted laser desorption ionization-time of flight mass spectrometry to characterize the serum peptide profile of HCC patients with bone metastasis. Indeed, a diagnostic model was determined using a learning algorithm of radial basis function neural network which identified a serglycin-derived peptide as one of seven peptides which may be utilized as a diagnosis tool for HCC metastasis to bone (23). Furthermore, the usage of quantitative tissue proteomics analysis indicated versican as a promising biomarker for the detection of HCC at an early stage (58).

Genome-wide methylation analysis of bladder cancer tissues discovered hypermethylation in the promoter region of a number of genes; with the combined hypermethylation of SOX1, PITX2, or versican identifying patients with a higher risk of bladder cancer morbidity (59). The utilization of microarray expression profiling on a group of 84 matched clear cell renal carcinoma and normal renal tissues determined a higher expression of the hyalectan, versican in tumor tissues. This was positively correlated to metastasis and worse prognosis (60).

Furthermore, upon applying an unsupervised method of data analysis, using singular value decomposition to microarray data sets of ovarian cancer tissues, a total of 151 targets was identified. Evaluation of the selected target genes by Real-time PCR showed that dermatan sulfate PG3 and LOX were strongly associated with overall as well as with disease-free survival (61).

Upon utilizing multiple microarray cohorts for genome screening, especially focused on genes correlated to disease relapse in stage II-III colon cancer patients, stromal versican expression was identified as a biomarker (62). When the Cancer Genome Atlas data set of 540 glioblastoma patients was analyzed with a R2 analysis webtool (R2 Genomics and Visualization Platform, Oncogenomics, AMC) and correlated to matching transcriptome and survival data their serglycin expression was shown to be grade dependent as well as positively associated with tumor tissue infiltration by mast cells (63).

Recently, Brézillon et al., have demonstrated that Raman micro-spectroscopy allows recording of the discrete GAG profiles of individual live cells making feasible its use for cell screening purposes. This method can potentially be utilized for identifying specific molecular signatures of GAGs as a marker of cancer progression in tissues (64). This is important as during tumor progression there is a remodeling of PG glycosylation of both cell surfaces and the ECM, among other, through heparanase action (65). Heparanase, is an enzyme that cleaves PG HS-side chains (66), thus strongly modulating various regulatory pathways, including the bio accessibility of HS-binding growth factors and cytokines (10). A clinical application of “glycan signature” is illustrated by a study showing that the type of PG glycosylation can affect the ability of immune cells to infiltrate tumor tissues and engage in the immune response (67).

However, previously well-established methods can likewise detect changes in PG molecular signature in some tumor types. Indeed, in urothelial bladder carcinoma the utilization of enzyme-linked immunosorbent assays (ELISA) showed that serum levels of SLRPs lumican, biglycan, and decorin were significantly altered in patients as compared with healthy controls (68). Additionally, the increased expression of lumican in urothelial cancer patients has been suggested as potential non-invasive marker for early detection of bladder cancer (68). Moreover, measurement of shedded syndecan-1 serum levels by ELISA in prostate patients revealed a correlation of higher levels with advanced cancer stage as well as with adverse overall survival and DSS in a multivariable pre-operative model. These findings suggest that the evaluation of sSDC1-levels is a promising tool for pre-operative risk-stratification in this group of patients (69).

Potential utilization of PGs as markers for therapy evaluation has also been proposed. Thus syndecan-4 mRNA expression has been indicated as a novel marker for the prediction of glioblastoma multiforme patient’s response to treatment with the WT1 peptide vaccine (70). Furthermore, an increase in plasma glypican-1 positive exosomes and a reduction in plasma miR-96-5p and miR-149 expression were correlated to colorectal cancer diagnosis; whereas a normalization of these markers’ levels was achieved after successful colorectal cancer surgery. The exosomes were isolated by ExoCapTM Exosome Isolation and Enrichment kit and analyzed by transmission electron microscopy and flow cytometry. Through this approach, glypican-1 positive exosomes as well as plasma miR-96-5p and miR-149 are suggested as markers for colorectal cancer diagnosis and therapy evaluation (71) Furthermore, in the case of colorectal cancer patients with peritoneal metastases, it was found that high epithelial versican expression in combination with high vascular endothelial growth factor levels were markers of better response to cytoreductive surgery and hyperthermic intraperitoneal chemotherapy, resulting in higher overall survival (72). PG expression and mechanisms of action in cancer are summarized in Table 1.

**Table 1:** Expression of proteoglycans (PGs) in tumor tissues and role in cancer pathogenesis.

PG	Class	Expressed by tumor tissue	Cancer cell function affected
Serglycin	Intracellular	Lung, colon, breast, prostate cancer (21), hematological cancer (20), nasopharyngeal cancer (22), hepatocellular cancer (23)	↑ Cancer growth (21)Hematological cancer malignant transformation (20)↑ Nasopharyngeal cancer metastasis (22)↑ Hepatocellular cancer metastasis to bone (23)

Perlecan	Basement membrane	Liver tumor blood vessels (26), melanoma (27), tumor blood vessels	↑ Melanoma invasion (28)↑ Melanoma growth and angiogenesis (29)

Versican	Pericellular matrix	Prostate, breast, glioblastoma, laryngeal, ovarian, pancreatic, cervical, gastric cancer (30)	↑ Breast cancer growth, mobility and metastasis^54^↓ Poor breast cancer response to chemotherapeutic drugs (54)

Aggrecan	Pericellular matrix	Laryngeal cancer (32)	↓ Laryngeal cancer metastasis (32)

Decorin	Extracellular matrix (ECM) proper	Tumor stroma (33)	Tumor suppressor (33)

Lumican	ECM proper	Breast, osteosarcoma, colorectal, prostate, pancreatic, lung, cervical cancer (7)	Role dependent on expression and tumor type (7)

Biglycan	ECM proper	prostate, colorectal, melanoma, pancreatic, bladder cancer (34)	Tumor promoter (34)

Syndecan-2	Cell membrane	Fibrosarcoma, osteosarcoma, breast, lung cancer (10, 18)	Role dependent on tumor type (10, 18)

Syndecan-1	Cell membrane	Multiple myeloma (43)	↑ Multiple myeloma growth and viability (43)

Glypican-1	Cell membrane	Colorectal cancer	↓ Metastasis

GLypican-3	Cell membrane	Hepatocellular cancer (36)	Cell-cycle arrest at G1 phase (36)

## PGs as Therapeutic Targets in Cancer

The idea of using PGs as therapeutical targets in cancer dates more than 30 years as Real et al in 1985 identified CSPG4 as a specific surface antigen of melanoma cells (73). This early report was followed by numerous studies performed by cancer research-oriented groups which resulted in multilevel approaches targeting PGs during cancer progression. Indeed, as recently discussed by Ilieva et al., CSPG4 “has been associated with the pathology of multiple types of cancer such as melanoma, breast cancer, squamous cell carcinoma, mesothelioma, neuroblastoma, adult and pediatric sarcomas, and some hematological cancers” and has become the goal of different, under development, therapeutical strategies (74).

Recently, cancer-selective tetra-branched peptides, with the ability to bind with high specificity to the GAG components of HSPGs have been developed. These peptides’ chemical structure allows coupling with various functional units utilized in cancer therapy. Noteworthy, peptides bearing methotrexate were found to by-pass the obtained, by breast cancer cells, resistance to the drug (75). The HSPG glypican-2 was recently shown to be highly expressed in neuroblastoma but not detectable in normal childhood tissues. Moreover, Bosse et al. produced an antibody-drug conjugate directed against glypican-2 with a high cytotoxic potential for neuroblastoma cells (76). Another, promising approach employs the putative immunomodulatory effects of PGs. Thus, proteolysis of versican deposited to colorectal cancer microenvironment resulted in the generation of novel matrikines and facilitated the infiltration of T cells to tumor tissues (77). A recent, preclinical proof of concept study shows that targeting PGs abundant in chondrosarcoma ECM results in a significant enhancement of the therapeutic index (78).

Even though, a large pool of data on PGs roles in cancer has been obtained limited progress has been made in translating basic research to clinical practice. The up to date performed clinical trials have focused on PG-directed vaccines. Patients with advanced HCC and high expression of GPC3 exhibited increased survival as compared to patients with low tumor GPC3 expression upon treatment with a novel recombinant humanized monoclonal antibody against GPC3 (79). Likewise, in two separate studies temporary disease stabilization was achieved after treating HCC patients with a peptide vaccine against GPC3 (80, 81). Moreover, blockade of the programmed death-1/PD-L1 pathway in combination with a GPC3 vaccine increased the immune response of vaccine-induced T lymphocytes in HCC patients (82). Furthermore, treatment of two recurrent ovarian clear cell carcinoma patients with a GPC3-derived peptide vaccine resulted in the stabilization of disease for over 1 year (83) (Table 2).

**Table 2:** Proteoglycans (PGs) as biomarkers and/or therapy targets.

PG	Biomarker/therapy target	Tumor type
Serglycin	– Unfavorable marker of metastasis-free disease and disease-free survival (22) – Mast cell recruitment (63) – Theranostic target (22) unfavorable marker of metastasis (23)	– Nasopharyngeal cancer (22) – glioblastoma (63) – Nasopharyngeal cancer (22) Hepatocellular cancer (23)

Versican	– Early disease marker (58) disease relapse marker (62)	Hepatocellular cancer (58) colon cancer (62)

Aggrecan	– Metastasis marker (32)	Laryngeal cancer (32)

Decorin	– Serum levels are a disease marker (68)	Bladder cancer (68)

Lumican	– Serum levels are a disease marker (68)	Bladder cancer (68)

Biglycan	– Serum levels are a disease marker (68)	Bladder cancer (68)

Syndecan-1	– Shedded syndecan-1 serum levels are of advanced cancer stage and adverse overall survival (69)	Prostate cancer (69)

Syndecan-4	– mRNA expression is marker of response to therapy (70)	Glioblastoma (70)

Glypican-1	– Positive exosomes are cancer diagnosis and therapy response marker (71)	Colorectal cancer (71)

Glypican-3	– Target for peptide vaccine (79–83)	Hepatocellular cancer (79–83) ovarian cancer (83)

Glypican-2	– Target for antibody-drug conjugate (76)	Neuroblastoma (76)

## Conclusion

The evidence that PGs have a key role in pathogenesis of cancer has led to the conclusion that understanding the changes in PG expression, fine structure as well as localization may lead to the development of innovative biomarkers and selective, more efficient therapies.

## Author Contributions

DN participated in drafting, writing, and editing the manuscript. AB and IS participated in writing and editing the manuscript. TK participated in writing and illustration preparation. AT and GT participated in drafting and editing the manuscript.

## Conflict of Interest Statement

The authors declare that the research was conducted in the absence of any commercial or financial relationships that could be construed as a potential conflict of interest.
